# Bioresponsive Hydrogels: Chemical Strategies and Perspectives in Tissue Engineering

**DOI:** 10.3390/gels2040028

**Published:** 2016-10-14

**Authors:** Antonella Sgambato, Laura Cipolla, Laura Russo

**Affiliations:** Department of Biotechnology and Biosciences, University of Milano-Bicocca, 20126 Milano, Italy; antonella.sgambato@unimib.it

**Keywords:** hydrogels, tissue engineering, bioconjugation

## Abstract

Disease, trauma, and aging account for a significant number of clinical disorders. Regenerative medicine is emerging as a very promising therapeutic option. The design and development of new cell-customised biomaterials able to mimic extracellular matrix (ECM) functionalities represents one of the major strategies to control the cell fate and stimulate tissue regeneration. Recently, hydrogels have received a considerable interest for their use in the modulation and control of cell fate during the regeneration processes. Several synthetic bioresponsive hydrogels are being developed in order to facilitate cell-matrix and cell-cell interactions. In this review, new strategies and future perspectives of such synthetic cell microenvironments will be highlighted.

## 1. Introduction

Bioresponsive hydrogels are dynamic systems that are capable of responding to or stimulating specific signals through the natural biological processes [1]. The possibility to tailor hydrogel composition, stiffness, and degradation rate makes this class of materials promising tools for tissue engineering applications. Hydrogels are cross-linked 3D networks containing hydrophilic polymer chains able to adsorb a significant amount of water [2] with a high versatility degree of chemical and physical properties. In addition, hydrogels in vivo administration can be performed by minimally invasive methods in order to avoid complex surgical intervention, even in irregular target sites of injured tissues [2]. Among these, bioresponsive hydrogels are considered “smart” biomaterials and are attracting great interest thanks to their controllable physical and biochemical properties when exposed to specific conditions in our body such as temperature, pH, or enzymes and receptors [3]. These general features may result in hydrogel responses (i.e., in term of swelling, degradation, mechanical deformation) [4] and/or in cells and tissue responses. This ability can be obtained and controlled by the spatial functionalization of hydrogel constituents with specific biological entities (biocues) in order to induce the desired stimuli [5]. Biological entities used with these aims can be native or synthetic biomacromolecules, such as enzymes, antibodies, nucleic acids [6], or small bioactive molecules such as carbohydrates [7] or peptides [8]. In this review, we will focus on a brief overview of the strategies employed to obtain bioresponsive hydrogel through functionalization with bioactive molecules.

## 2. Classification of the Hydrogels

**Figure 1:**
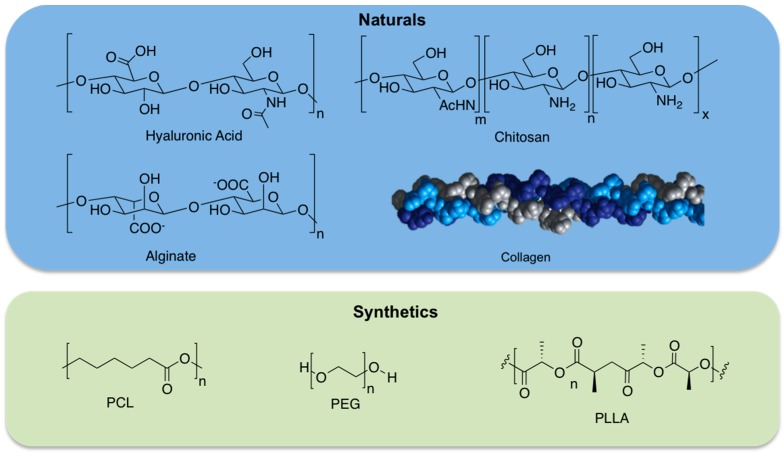
Chemical structure of some natural, and synthetic hydrogels.

On the basis of their composition, it is possible to distinguish natural, synthetic, and composite or hybrid hydrogels (Figure 1).

### 2.1. Natural Hydrogels

Natural hydrogels are made up of natural biomacromolecules including proteins and polysaccharides. These biomacromolecules have different natural origins (animals, plants, or microorganisms). Several polysaccharide-based or protein-based hydrogels have been synthesized. A number of examples of polysaccharide-based hydrogels with tuneable properties are available for cartilage and bone tissue engineering applications. Most cited hydrogels for these applications have been prepared using hyaluronic acid [9], chitosan [10], and alginate [11] biopolymers. Hyaluronic acid (HA), alginate, and chitosan are established examples of polysaccharides used in tissue engineering applications. It is known that HA is a non-sulphated glycosaminoglycan (GAG) ubiquitous in mammalian tissues. It is a linear polysaccharide composed of a repeating disaccharide of (1–3) and (1–4)-linked β-d-glucuronic acid and *N*-acetyl-β-d-glucosamine units, overproduced during the wound healing process.

HA-based hydrogels have been produced by covalent cross-linking, for example, by hydrazide derivatives, by esterification, by carbodiimide chemistry, and by Huisgen-type cycloaddition (click chemistry) [12]. Additionally, HA has also been combined with synthetic polymers, proteins, and peptides in order to produce hybrid hydrogels [13]. Chitosan has been employed for different tissue regenerative approaches. It is a polysaccharide made up of (1–4)-linked d-glucosamine and *N*-acetyl-d-glucosamine units derived from chitin, a constituent of arthropod exoskeletons, but its structure is similar to naturally occurring GAGs. One of the main chitosan properties is its solubility in diluted acids by protonation of the amino groups so that it can be gelled, for example, by pH increase. Chitosan hydrogels were also obtained via UV-assisted photopolymerization, via cross-linking agents (i.e., genipin, glutaraldehyde, and squarate), or thermal variations [14].

Alginates are composed of guluronic acid and mannuronic acid. Their abundance, and low prices, allow a widespread use in the food industry as thickeners, emulsifiers, and in tissue engineering applications. Alginate was also used for various biomedical applications, such as drug delivery and cell encapsulation, because it is able to gel under mild conditions by the addition of divalent cations, it is biodegradable, and has low toxicity. Alginate-based hydrogels have also been obtained by chemical cross-linking by adipic hydrazide or PEG using carbodiimide chemistry [15].

Thanks to their structural role in nature, also protein-based hydrogels gained great interest for tissue engineering [16]. Collagen-based hydrogels have found application due to its ubiquitous presence in different tissues of the human body. Several cross-linking strategies were performed to control mechanical properties and 3D structures using for example 1,4-butanediol diglycidyl ether (BDDGE) or genipin cross-linking [17,18]. Another protein, gelatin (obtained from collagen hydrolysis) has captured increasing attention as it has relatively low antigenicity although maintaining the properties of biocompatibility and biodegradability; moreover, gelatin is significantly less expensive than collagen [19]. Several examples of photocrosslinked gelatin (i.e., gelatin methacrylamide (GelMA)) with tunable physico-chemical and biological properties were investigated for tissue engineering applications [20,21]. Other chemoselective cross-linking strategies involve thiol-ene photopolymerization between thiolated gelatin and pentenoyl gelatin [22] or thiolated gelatin and PEGdA [23]. These are just a few examples of natural biopolymers used for tissue engineering applications.

### 2.2. Synthetic Hydrogels

Even though hydrogels derived from natural biomacromolecules, such as proteins or polysaccharides, can actively support cell viability or cell differentiation, these materials have poor mechanical features and they are hard to process, and it is also difficult to maintain product consistency. To overcome these limitations, synthetic materials represent promising starting materials in scaffold design [24]. In fact, synthetic hydrogels can be designed using polymers with controlled molecular weight and biodegradable linkers. These features allow for the fine tuning of hydrogel composition, formation dynamics, mechanical properties, and degradation rates. Examples of synthetic materials are poly(ethylene glycol) (PEG), poly(lactide) (PLA), and poly(ε-caprolactone) (PCL). PEG is a hydrophilic polymer, currently Food and Drug Administration (FDA) approved for several biomedical applications, and is one of the most frequently applied synthetic polymers for hydrogel preparation. Usually PEG acrylates or methacrylates can be photopolymerized, affording controlled hydrogel architectures [25]. Thermally reversible hydrogels have also been produced from block copolymers of the hydrophilic PEG and several polyesters as the hydrophobic block; PLA, poly(glycolic-*co*-lactic acid) (PLGA), PCL, and poly(l-lactic acid) (PLLA) are extensively used [26] because of their biocompatibility, biodegradability and facile synthesis by ring opening polymerization of lactide, glycolide, or ε-caprolactone monomers.

However, synthetic polymers lack cell adhesion sites and specific characteristics able to induce biological responses. In order to overcome these drawbacks, extensive conjugation chemistry has been applied to introduce bioactive molecules, such as the well-known RGD peptide (arginine-glycine-aspartic acid) to allow cell adhesion [27], or other molecules able to induce and guide specific biological phenomena (i.e., growth factors or carbohydrates able to guide cell differentiation) [28]. Different bioconjugation methods have been developed to introduce biological functions into synthetic materials [29].

## 3. From Tissue Complexity to Hydrogel Design: The Simplification Game

In the area of tissue engineering, hydrogels can be tuned in order to satisfy different design parameters for their integration in the damaged site, and, therefore, to operate in an appropriate manner and support new tissue formation. Hydrogel-based materials are able to supply a three-dimensional structure to adequately support cell proliferation, tissue integration, and differentiation. This tipology of 3D structure reproduces very closely the nature of tissues and takes into account the morphology and gene expression. In order to develop hydrogels for tissue engineering, different parameters must be considered, including mechanical and physico-chemical properties (i.e., biodegradation) and biological performance parameters (i.e., biocompatibility, cell adhesion, and proliferation). Moreover, it is important to consider the availability and commercial feasibility when producing hydrogels for tissue engineering applications. The native extracellular matrix (ECM) supplies a plethora of signals to neighbouring cells that modulate functional outputs, in combination with cell-cell and cell-ECM signalling [30]. Moreover, the extracellular matrix should be imagined as a dynamic milieu where the local environment can be reshaped through cell-mediated secretion and deposition of biomolecules or degraded through cell-secreted enzymes called matrix metalloproteinases (MMPs). In nature, the in vivo organization of tissues, with regard to their development and remodelling, is controlled by several factors that regulate and interplay at different levels in time and space. In tissue engineering and regenerative medicine studies, an adequate environment is necessary to supply the essential factors able to drive cell functions in vivo; in this way, it is possible to direct cells to differentiate in the right way [31]. Hydrogels made up of naturally derived components, for example extracellular matrix proteins (such as collagen) and polysaccharides like GAGs, have received particular attention in different applications in the field of regenerative medicine [5], since they supply physico-chemical and biochemical features that are similar to the native cellular milieu. Mimicking the natural tissue and microenvironment is not a simple objective; the simplification is possible thanks to the growth in the knowledge about signalling processes that regulate cell behaviour. To promote the understanding of the cell-niche interactions, hydrogels have been extensively employed as artificial cell niche given their tissue-like water content as well as tunable physico-chemical properties. However, only a few hydrogels produced to date allow the control of cell microenvironment properties such as biochemical signals and mechanical stiffness [32].

## 4. Bioactivation Strategies

Bio(macro)molecule conjugation (bioconjugation) is a useful approach that allows for the improvement of the hydrogels through signalling molecules, such as proteins, peptides [33,34], or even carbohydrate epitopes [35,36,37], in order to modulate and drive cellular fate. The conjugated biomolecules can be chosen in order to mediate different cellular events that are fundamental in regenerative medicine approaches, such as adhesion, migration, proliferation, and differentiation. Probably, the idea of bioresponsive hydrogel came from the pivotal study by Tirrell and co-workers [38] on self-assembly and gelation of a triblock artificial protein that was conjugated to a PEG moiety in order to control the gelling properties as a function of pH and temperature. Generally speaking, bioconjugated hydrogels may be classified into different groups depending on the bioactive molecule used for bioresponse activation: hydrogel-protein conjugates, hydrogel-peptide conjugates, hydrogel–glycan conjugates, and hydrogel-small molecule conjugates.

### 4.1. Hydrogel–Protein Conjugates

Proteins are fundamental and ubiquitous macromolecules playing key roles in the body mediating a plethora of cell processes and, for this reason, relevant for the design of hydrogel for tissue engineering approaches [39,40]. The incorporation of proteins and bioactive molecules in hydrogels allows for the control of the cellular microenvironment, and may be a valuable tool to support the regenerative process. However, their complex structure and, in some cases, the limited availability through biotechnology and molecular biology techniques may hamper the development of protein-conjugated hydrogels. Moreover, protein may have poor chemical stability and, together with their tendency to aggregate, their bioactivity may be limited. Despite these issues, various approaches have been developed to incorporate and deliver proteins into hydrogels. In general, different strategies can be used to include proteins into hydrogels, such as physical entrapment or covalent linkage to the hydrogel macromolecules. The bioconjugation through covalent bonds may offer some advantages in terms of stability against in vivo degradation forces and the maintenance of the therapeutic concentration in the hydrogel. A few examples of smart strategies for covalent protein bioconjugated hydrogels will be given in this section.

**Figure 2:**
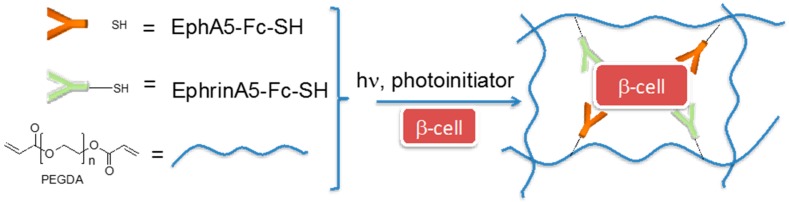
Schematic representation of poly(ethylene glycol) (PEG)-based hydrogels conjugated to EphA5-Fc and EphrinA5-Fc, tailored to sustain pancreatic β-cells survival.

Anseth and co-workers fabricated PEG hydrogels via a thiol-acrylate photopolymerization reaction; in particular, PEG-diacrylate precursors were conjugated to thiolated EphA5-Fc receptor and ephrinA5-Fc ligand for enhancing pancreatic β-cell survival (Figure 2) [41]. EphA receptor and ephrinA ligand are cell surface-bound proteins involved in, among other things, insulin secretion from pancreatic β-cells promoting cell adhesion and motility/morphology changes through the integrin signalling pathway. These bioresponsive hydrogels were shown to provide crucial cell-cell communication cues for cell survival and proliferation.

In other examples, transforming growth factor-β1 (TGF-β1) was linked to photopolymerizable methacrylated chitosan (MeGC) hydrogels [42] and to acrylated HA hydrogels [43]. The first hydrogel was successfully used to induce chondrogenesis in human mesenchymal stem cells (hMSCs), while the second one showed enhanced cell survival and engraftment of encapsulated murine cardiac progenitors to the host tissue after transplantation, accompanied by vascularization.

Toward cardiac tissue regeneration, several hydrogels functionalised with different or multiple growth factors have been proposed. For example, stromal derived factor-1α (SDF-1α) conjugated to a PEG hydrogel [44] in vivo demonstrated a sustained colonization of progenitor cells in the ischemic tissue and promoted the angiogenetic process. Similarly, the recruitment of progenitor cells induced re-epithelization and revascularization with alginate-based hydrogels bioconjugated to SDF-1α [45]. Another hot issue in regenerative medicine is neural tissue regeneration [46,47]. In this respect, an increase of axon outgrowth of dopaminergic neurons, from rat embryos or differentiated from stem cells in culture, was obtained through an injectable PEG-silica composite hydrogel conjugated to semaphorin 3A. Semaphorins are a class of secreted and membrane proteins particularly relevant in neural system development; they are involved in axonal growth cone guidance and in the deflection of axons from inappropriate regions. However, the presence of non-degradable silica particles embedded in the hydrogel resulted in an increase of macrophages and glial cells in long term implantation studies.

**Figure 3:**
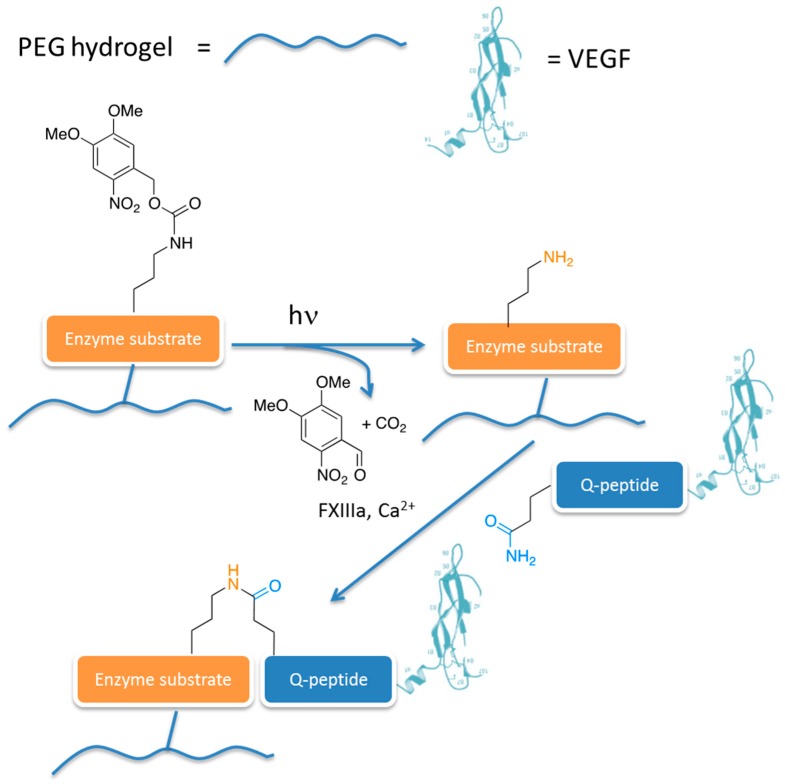
Strategy for the synthesis of PEG hydrogels conjugated to vascular endothelial growth factor (VEGF), through an enzyme-catalysed bioconjugation step, proposed to support primary human mesenchymal stem cells growth.

In general, the chemical bioconjugation of complex signalling proteins is a hard task, since the activity and stability of proteins can be compromised by the conjugation reaction that might not be sufficiently mild and/or chemoselective. In order to address this issue, an extremely interesting approach was proposed by Lutolf and co-workers, who obtained the spatiotemporally controlled enzyme-mediated bioconjugation of vascular endothelial growth factor 121 (VEGF_121_) and the recombinant fibronectin type III repeat 9-10 fragment (FN_9-10_) [48]. Briefly, the researchers synthesised a PEG-based hydrogel functionalised with a “masked” enzyme substrate that could be rendered accessible to the related enzyme by a photocatalysed reaction (Figure 3). Once the substrate is accessible, the enzyme (that is FXIIIa, a key enzyme involved in the blood coagulation cascade) catalyses the reaction between the ε-amino group of lysine (K) and the carboxyamide side chain of glutamine (Q) to the corresponding ε-(γ-glutamyl)lysine isopeptide. Using recombinant VEGF_121_ engineered with an exogenous peptide domain at the *N*-terminus containing the required glutamine to enable enzymatic cross-linking (Q-peptide in Figure 3), the enzymatic reaction affords the PEG hydrogel conjugated to the growth factor. The patterned PEG hydrogel is biocompatible for mesenchymal stem cells (MSC) cells.

### 4.2. Hydrogel–Peptide Conjugates

**Figure 4:**
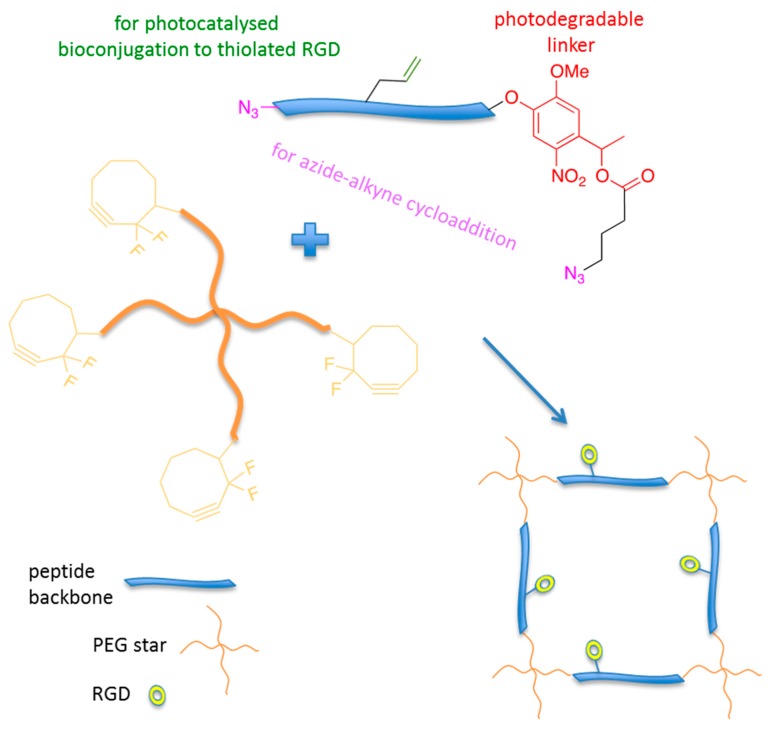
Strategy for the synthesis of photodegradable adhesive hydrogels for advanced 3D cell culture.

The bioconjugation of peptides in place of full-length proteins greatly simplifies the chemistry and may improve the efficacy of the bioconjugation. Since many mammalian cells are anchorage-dependent, cell adhesive properties of the hydrogel can be achieved by the introduction of small adhesive peptidic sequences into the hydrogel matrix [49]; for example, they can be derived from laminin, such as RGD (Arg-Gly-Asp) [50], LGTIPG, YIGSR, IKVAV, LRE, PDGSR, IKLLI, LRGDN [51], and from type I collagen and fibronectin, in other words, DGEA [52], KQAGDV, REDV, and PHSRN. PEG-based hydrogels are useful for tissue engineering applications due to their favourable porosity, mechanical properties, and biocompatibility. However, due to their chemical nature, PEG does not possess cell attachment motifs. The conjugation of adhesive peptides such as the RGD sequence improves PEG hydrogel biofunctionality, rendering PEG-based hydrogels more suitable mimics of ECM. In this respect, Anseth and co-workers synthesised a dynamically controlled PEG-based hydrogel containing the adhesive sequence RGD through a double-click reaction (Figure 4) [53].

**Figure 5:**
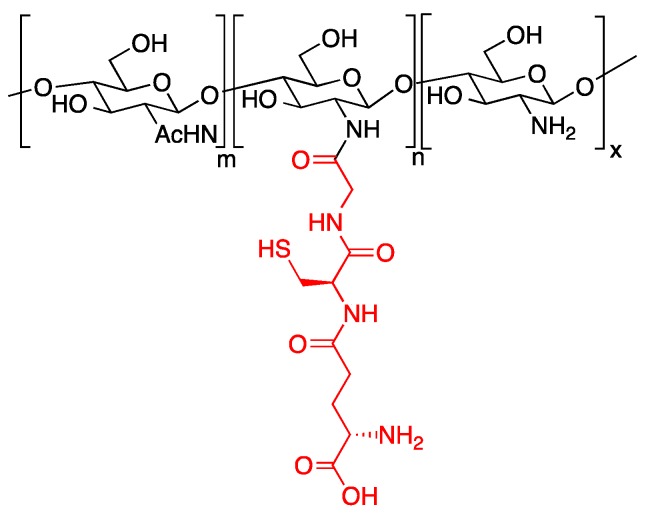
The chitosan-glutathione conjugated hydrogel able to suppress oxidative stress in cardiomiocytes.

The spacer length between adhesive peptides and the material scaffold may be a critical parameter for regulating cell phenotype in tissue engineering. An interesting example of this issue was given with alginate hydrogels functionalised with RGD peptides linked through varying spacer arm lengths and assessed with primary human fibroblasts either on 2D or 3D scaffolds [54]. Alginate hydrogels were functionalised with G_n_RGDSP moieties (n = number of glycine units) through EDC/sulfo-NHS coupling. Four glycine units in the spacer arm is essential for enhanced adhesion and growth of fibroblasts. An optimal spacer length was also needed for minimizing cellular stress, as determined by the expression of heat shock proteins and Bcl-2. Despite the extensive use of adhesive sequences, such as RGD and close peptides, other amino acid sequences have been conjugated to hydrogels in order to stimulate different cellular responses. For example, in order to control and reduce the oxidative stress experienced by cardiomiocytes (CMs) in myocardial infarction, the antioxidant tripeptide glutathione was bioconjugated to a chitosan-based hydrogel (Figure 5) [55]. The glutathione-conjugated chitosan is effective in vitro as an antioxidant, is biocompatible in the presence of cardiac myocytes, and is able to suppress the oxidative damage and apoptosis in CMs by removing the excessive intracellular reactive oxygen species (ROS) content.

Still in cardiac tissue engineering, the peptide sequence QHREDGS, derived from the fibrinogen-like domain of angiopoietin-1, was conjugated to a collagen-chitosan hydrogel. Peptide-modified hydrogels induce the tube-like structure formation in encapsulated endothelial cells [56].

**Figure 6:**
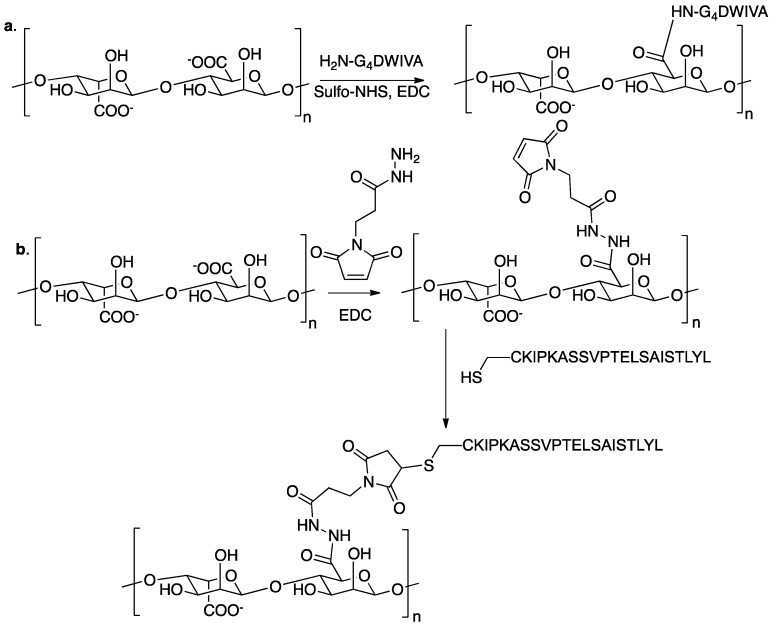
Alginate-based hydrogel designed to promote osteogenesis in murine mesenchymal stem cells. (**a**) Conjugation strategy for the DWIVA peptide; (**b**) Conjugation strategy for the “knuckle epitope”.

In order to promote osteogenesis, alginate hydrogels bioconjugated to peptide mimics of BMP-2 were studied with encapsulated osteoblasts and mesenchymal stem cells. Two different peptide sequences derived from bone morphogenetic protein 2 (BMP-2) were incorporated into the alginate backbone by two different chemoselective strategies that could guarantee the spatial orientation of the peptides in their active form. The peptide DWIVA was covalently bound to alginate by carbodiimide chemistry through a four glycine spacer (Figure 6a) [57], while the so-called “knuckle epitope” of BMP-2 (KIPKASSVPTELSAISTLYL), was modified with a cysteine residue at the *N*-terminal end; the thiol group was conjugated via a Michael addition to the alginate modified with maleimide groups (Figure 6b).

Alginate functionalised with the knuckle epitope was shown to increase alkaline phosphatase activity in clonally derived murine osteoblasts, while with clonally derived murine mesenchymal stem cells it initiated Smad signalling, up-regulated osteopontin production, and increased mineral deposition.

Alginate-based hydrogels were also bioconjugated to the YIGSR peptide through amide bonds to the carboxylic acid groups of the alginate. The peptide-modified alginate hydrogels allowed adhesion of NB2a neuroblastoma cells and promoted neurite outgrowth [58]. The adhesion of NB2a neuroblastoma cells and neurite outgrowth was found to be a function of the peptide density.

### 4.3. Hydrogel–Glycan Conjugates

Thanks to their unique properties in signalling and cell development, glycans represent an interesting class of molecules to incorporate in hydrogels to make them bioresponsive. There are several examples of glycan epitopes used to bio-activate natural and synthetic materials [7]. In hydrogel systems glycan conjugates have been used in order to control both cell growth and vitality [59,60]. Heparin possesses binding domains to many growth factors, hence, when included in hydrogel design it may be a useful tool to allow for the retention and subsequent delivery of growth factors [59]. This approach was used to promote angiogenesis. In more detail, tyramine was first introduced into the gelatin backbone as the cross-linking points, then heparin was covalently linked to gelatin-tyramine. Vascular endothelial growth factor (VEGF) was then incorporated into the gelatin derivative by non-bonding interactions with heparin binding motifs and finally enzymatic reactions with hydrogen peroxide (H_2_O_2_) and horseradish peroxidase (HRP) allowed the formation of the gel by oxidative cross-linking [61]. In vivo implantation experiments showed deeper and denser cell infiltration and angiogenesis in the heparin-modified gelatin/VEGF gels than in the controls.

Long-term in vitro maintenance of primary hepatocytes is a burden for hepatic tissue engineering, since these cells lose their phenotype in standard culture conditions. Foster et al. studied the use of heparin-containing hydrogels as scaffolds for the culture and for the maintenance of functional primary hepatocytes [60]. Thiolated heparin was conjugated to diacrylated PEG via a thiol-ene photocatalysed reaction. Analysis of hepatic functionality of rat hepatocytes cultured on the hydrogel revealed that cells sustained albumin secretion for at least three weeks and increased cytochrome P450 activity. In addition, hepatocyte growth factor (HGF) was also entrapped into the gels thanks to heparin interactions; in the presence of HGF, higher amounts of albumin could be observed.

### 4.4. Hydrogel–Small Molecule Conjugates

Promising approaches in tissue regeneration rely on both suitable scaffold design and on the application of stem cells and the development of efficient approaches to control their fate. Recently, several small molecules have been identified as able to induce stem cell differentiation both in vitro and in vivo [62]. Within this frame, several hydrogels conjugated to small molecules, either to promote gelation or to impart selected biological stimuli, have been synthesised.

**Figure 7:**
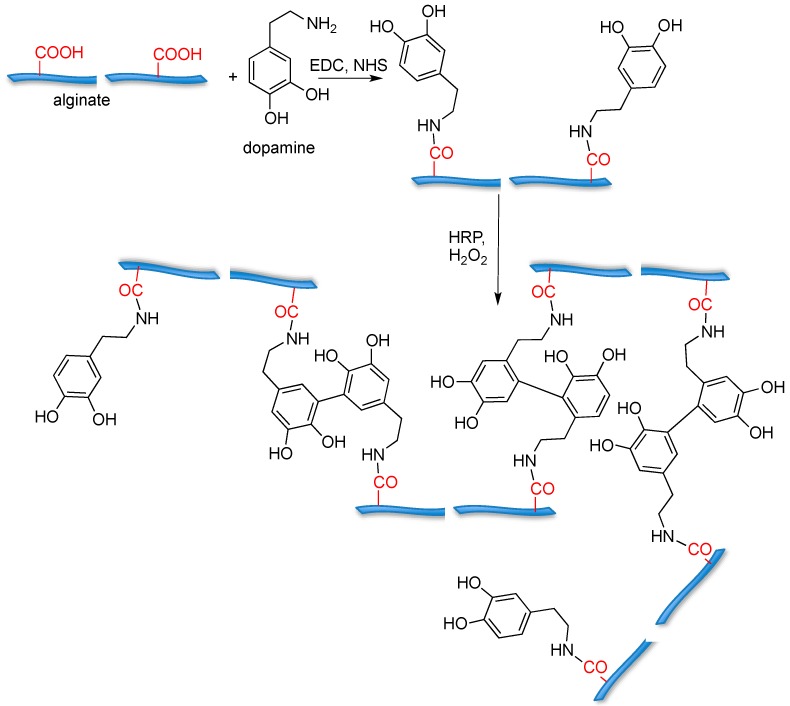
Alginate-based hydrogels cross-linked through dopamine oxidation by horseradish peroxidase (HRP); biocompatibility was assayed with NIH 3T3 cells.

For example, tyramine [63,64] and dopamine [65] conjugated alginate have been prepared in order to induce gelation through enzymatic oxidation; dopamine was conjugated to alginate (Figure 7) and subsequently gelled by enzymatic cross-linking mediated by horseradish peroxidase (HRP) and H_2_O_2_.

The resulting hydrogels are cytocompatible when assayed with NIH 3T3 cells. In addition, compared with unfunctionalised alginate hydrogels, the dopamine-conjugated hydrogel showed higher cell adhesion and elasticity properties.

Catechol moieties were also grafted onto chitosan by reductive amination, and the resulting macromers were gelled through coordinative interaction with transition metal ions, such as iron, or in oxidative conditions by NaIO_4_ [66].

Chitosan–catechol conjugates were also used as precursors for the synthesis of hybrid hydrogels in the presence of thiolated Pluronic F-127 triblock copolymer; the resulting hybrid material showed temperature-sensitive and adhesive properties to mucous layers and to soft tissues, together with good hemostatic properties useful for wound healing [67].

Other small molecule motifs have been proposed for the formation of biofunctional supramolecular hydrogels, such as bisphosphonates or glucosamine, that showed wound-healing properties [68].

Small molecules can be grafted onto hydrogel macromers in order to induce a specific cellular response or to promote cell differentiation. Bisphosphonate moieties have been grafted onto hyaluronic acid hydrogels in order to promote BMP-2 sequestration and bone regeneration [69]. In more detail, hyaluronic acid precursors were covalently functionalised with bisphosphonate ligands; the BP moieties are efficient sequestering agents of BMP-2 that can be protected from degradation and released in the site of administration by enzymatic hydrolysis by hyaluronidases.

Biological studies showed that BMP-2 entrapped in hyaluronic acid-bisphosphonates hydrogel maintains its bioactivity, as shown by the induction of osteogenic differentiation of mesenchymal stem cells.

A similar approach toward bone tissue regeneration was proposed by Furukawa and co-workers [70], who developed a hyaluronic-based hydrogel functionalised with inorganic polyphosphate moieties. The poly-phosphates groups were able to provide osteoconductive stimulation to murine osteoblast precursor cells, demonstrated by the up-regulation of osteogenic marker genes and increased alkaline phosphatase activity. In addition, it was shown that the bioactivity imparted by immobilised pyrophosphates was higher if compared to free polyphosphates embedded within the gel.

Dexamethasone, a synthetic corticosteroid, was also used as a small-molecule for the stimulation of osteogenic differentiation [71,72]. Thus, PEG-based hydrogels were functionalised with dexamethasone through a Diels-Alder conjugation strategy. The reversible nature of the Diels-Alder reaction was exploited for controlling dexamethasone release from the hydrogel. Both in 2D and in 3D hMSCs cell culture, dexamethasone release promoted a significant increase in alkaline phosphatase activity and mineral deposition if compared to that of control gels without dexamethasone, or with dexamethasone in the free form.

## 5. Outlook and Perspectives

The field of hydrogels started with the pioneering Wichterle and Lim in the 1960s [73,74]. Since then, a remarkable development of hydrogels from simple chemically or physically crosslinked networks to complex bioresponsive systems was observed. Although not cited in this review, hydrogels are gaining a high level of sophistication, reflected, for example, in shape memory and self-healing hydrogels [75,76]. The clinical need for easy administration in regenerative medicine applications fuelled the research of injectable hydrogels and bioresponsive constructs able to drive cell response suitable for minimally invasive treatments [77]. The selection of the cross-linking strategy is driven by the need of an immediate change from a low viscous solution before injection and quick formation of a strong network in situ. In addition, the possibility to modulate the degradation profiles after hydrogel administration and the bioactivation strategy can further improve the clinical translation of these scaffolds for tissue engineering applications. It is expected in the next years that more sophisticated hydrogels suitably tuned to sustain and promote adhesion, migration, and differentiation of specific cell lines will be created, bringing cell therapies in tissue regeneration closer to clinical application.
